# Role of green finance, green bonds, public private partnership, and technology innovation in carbon neutrality and sustainable development

**DOI:** 10.1016/j.heliyon.2024.e37189

**Published:** 2024-09-03

**Authors:** Yiran Zhang

**Affiliations:** School of Economics, University of Queensland, Brisbane, Australia

**Keywords:** Green finance, Green bond, Carbon neutrality and sustainable development

## Abstract

In recent years, global attention has increasingly turned towards the urgent goal of eliminating carbon emissions. Evaluating new methods may be the key to achieving environmentally responsible growth. This study examines the impact of China's public-private energy partnerships on CO_2_ emissions from 1990Q1 to 2022Q4, employing Dynamic Ordinary Least Squares (DOLS) and fuzzy multi-objective least squares (FMOLS) econometric regression methods. Our analysis incorporates sustainability efforts, renewable energy sources, environmentally sound finance, and technological advancements to provide a comprehensive understanding of CO_2_ emissions dynamics. Our findings reveal that expanding access to credit has facilitated the development of green financing, resulting in a favorable environmental impact of China's economic growth and public-private partnerships. The reduction in carbon dioxide emissions is counter balanced by the positive effects of technological progress, increased utilization of renewable energy sources, and enhanced power efficiency. We highlight the multiplying effect of these factors, underscoring the need for global public-private collaborations in the energy sector to significantly lower carbon emissions and to achieve sustainable development goals. We assert that policymakers tasked with facilitating China's transition to renewable energy sources must prioritize environmental preservation, technological advancement, and the optimal utilization of renewable resources to achieve lasting sustainability.

## 1 Introduction

To achieve longer-term, sustainable financial development without compromising efforts to protect the environment, widespread adoption of environmentally friendly and clean technologies is essential. Expanding the economy necessitates increased research, accelerated innovation within the sector, and a gradual transition to renewable energy sources [1]. Sustainable energy solutions in developing nations, where the impact of economic wealth and efficacy is less understood, are subject to ongoing research and management initiatives [2]. However, the growth of the renewable energy sector presents opportunities to address these issues. Academic research has extensively examined the driving factors of this industry, including technical advancement, structural and political support, and economic and social barriers [3].

Investing in renewable energy carries substantial risks due to high upfront costs and long payback periods associated with new technology. Therefore, access to stable funding sources is crucial for its development. Establishing a stable financial system can lead to reduced transaction costs, lower risks, more efficient resource allocation, and increased investment opportunities [4].

The global financial system's transition to digital platforms has intensified pressure on the financial industry to adapt. Blockchain technology, social networks, big data analytics, and artificial intelligence have significantly benefited the financial sector, resulting in lower costs, improved services, reduced information disparities, greater flexibility, and a more diverse and resilient system [5]. Introducing digital finance has propelled the financial services industry forward, prompting existing institutions to enhance their offerings to remain competitive. Customers may increasingly opt for digital funding over traditional lending methods, further driving this shift.

Given the financial challenges faced by the renewable energy sector, leveraging digital funding options could alleviate some of these difficulties. Developing alternative financing mechanisms could help solar power enterprises meet their financial needs. Simulated outcomes suggest that using digital finance for renewable energy production could have positive environmental impacts while remaining within resource constraints. The rise of digital economics has filled gaps left by traditional finance, offering effective solutions for businesses' financial needs and freeing up resources for innovation [6].

The objectives of this are as under.

(1) To analyze the impact of cooperative initiatives between public and private organizations in China's energy industry on carbon dioxide emissions from the first quarter of 1990 to the fourth quarter of 2022.
(2) To analyze the effect of increasing credit availability on the growth of green finance and its impact on the reduction of carbon emissions and to emphasize how decreases in carbon dioxide emissions caused by programs like green funding are counteracted by other variables such as technical advancements, greater use of renewable energy sources, and enhanced power efficiency.
(3) Highlight the Significance of cooperation between communities and commercial organizations in the energy sector to accomplish substantial reductions in carbon emissions and to provide insights into the intricate interaction between public and private entities.

This paper makes a significant contribution to existing research by offering both real-world evidence and methodological insights into how China's public-private energy partnerships are influencing CO_2_ emissions. Through the use of advanced statistical methods like DOLS and FMOLS, the study conducts a thorough examination of the intricate interplay between economic growth, the adoption of renewable energy, and environmental sustainability. This methodological approach not only bolsters the credibility of the findings but also establishes a model for future studies in this area.

Moreover, this study explores the impact of green digital finance on sustainability Key Performance Indicators (KPIs) from a limited-resources perspective, examining how promoting universal access to digital banking could help combat global warming. The research framework covers both transmission techniques and occurrence assumptions, contributing valuable insights to the field. It proposes a new strategy for achieving low-carbon green development and underscores the benefits of inclusive digital funding. The analysis employs mediation effects to describe how digital finance influences financial leverage and risk, budgetary constraints and expenditures, technological progress, and social dynamics. By integrating inclusive digital finance with ecological stewardship, the study aims to better understand their relative importance and optimize their synergy.

In conclusion, the study presents a comprehensive examination of the role of digital finance in promoting sustainable development, providing valuable insights for policymakers, researchers, and practitioners alike. Our study holds significant importance in the context of addressing the pressing global issue of carbon emissions reduction. By analyzing the impact of China's public-private energy partnerships on CO_2_ emissions, we contribute to the understanding of effective strategies for promoting environmental sustainability and economic growth simultaneously. This study is particularly relevant given the increasing focus on transitioning to renewable energy sources and reducing greenhouse gas emissions globally. Moreover, the research carries substantial policy implications for both China and the global community at large. By showcasing the positive environmental outcomes associated with increased access to financing for green initiatives and the promotion of renewable energy sources, the findings offer valuable guidance for policymakers and industry professionals striving to steer towards carbon neutrality and sustainable development objectives. By emphasizing the symbiotic relationship between economic advancement and environmental preservation, the study underscores the necessity of holistic strategies to combat climate change and emphasizes the vital role of collaboration between public and private entities in driving significant environmental progress.

The rest of the paper will be structured as follows: Section 2 provides a review of the supporting literature, Section 3 includes methodology and data, Section 4 contains results and analysis, and Section 5 concludes the paper with a short summary of findings, policy implications, and limitations of the paper.

## 2 Literature review

This study contributes to both the existing literature on financial constraints and the literature on financing renewable resources. Many influential works in the literature on investment in renewable resources focus on carbon emission constraints, including cap-and-trade legislation, coal subsidies, and carbon taxes. Publications on green finance and green trade credit exemplify the literature on financial constraints [7]. Dynamic manufacturing models developed by Refs. [8,9] have been applied in uncertain markets, forming the basis of much research on financing renewable energy. These models highlight the trade-off between lower emissions and higher initial investments required for green manufacturing practices. Businesses producing emissions-related goods and services must maintain a positive quota account balance and can engage in forward contract trading of quotas on external marketplaces, thereby reducing estimated discounted costs [10]. Studies have also investigated the impact of pricing rules on investment in renewable resources. Authors in Ref. [11] evaluated how carbon emission limits affect supply chains for fresh food, demonstrating that cap-and-trade systems can effectively balance business and environmental goals. Additionally, authors found that subsidies for traditional energy sources can limit investments in renewable energy.

Authors in Ref. [12] examined the environmental impact of the automobile industry, suggesting that comparing ecological performance among similar businesses and adopting a unified strategy for measuring and improving environmental performance can lead to steady operational enhancements. Using panel data [13], demonstrated the potential of renewable energy to mitigate carbon emissions. Similarly [14], found that strict ecological regulations promote green innovation in renewable energy machinery. Other studies offer further reading on carbon limits and renewable energy financing [15,16]. Authors in Ref. [17] analyzed the market impact of green sukuk issuance in Malaysia, while [18] studied corporate financing in China's energy-saving enterprises. Lastly [19], investigated the impact of economic growth and green technologies on carbon emissions, highlighting the importance of renewable energy adoption. Several other studies address the challenges and strategies of green financing [20,21].

The Environmental Footprint (EFP) quantifies the ecological consequences of human actions, and its increasing levels in developing nations emphasize the pressing necessity to identify and address the root causes of inadequate environmental conditions [22]. The correlation between energy consumption and environmental sustainability has been a central focus of research, particularly within the framework of the Environmental Kuznets Curve (EKC). The relationship has conventionally been portrayed as an inverted U-shaped curve. Recent research has examined intricate patterns, such as the N-shaped curve, to reveal a more nuanced connection between economic growth and environmental quality [23].

A comparison of this study with different studies is given in Table 1. Effectiveness throughout the supply chain is the primary focus of this strategy. Eco-friendly financing is available, and manufacturers may apply through financial institutions. According to the results of their research, the government may issue various carbon emission regulations to aid the supply chain members in identifying solutions that are beneficial to all parties involved. To investigate how the cap-and-trade system affects public utility firms' choices between investing in conventional and renewable energy sources [24], developed a model. According to their simulations, utility firms would put more money into renewable energy sources under the grandfathering model or the benchmarking mode than they would under the no cap-and-trade scenario. In the end, they came to this decision. Authors in Ref. [25] studied several Chinese economic variables to determine their interrelationships. Renewable Energy Investment (REI), green financing (GF), and renewable energy output (REOP) are some examples of these metrics. Based on their empirical results, strategies that aim to raise the volatility of IRE, GF, and REOP while concurrently strengthening asset in these subdivisions may help to achieve maintainable financial development, ecological sustainability, and renewable vigor manufacture [26].

**Table 1:** Instant of maximum connected investigation: comparisons and modifications.

Education	Cap-and-trade	Green economics	Conservative reserve	Renewable supply
[27]			✓	✓
[28]		✓	✓	
[29]	✓		✓	✓
[30]		✓		✓
This study	✓	✓	✓	✓

In contrast to the studies mentioned above, the current research uses three separate models to examine the effect of green finance on the use of renewable resources within the framework of a cap-and-trade system. This study also examines how the interest rate on green loans and the company's initial working capital affect the choice between conventional and renewable power production. The study seeks to address various potential research gaps based on the objectives stated in the paragraph. There exists a lack of awareness of the precise influence of public-private energy partnerships on carbon emissions. This study aims to address this knowledge gap by examining the intricacies of these interactions and their environmental ramifications. Previous studies have generally concentrated on isolated factors that impact carbon emissions, such as the adoption of renewable energy or technical advancements, without taking into account their collective impact. The objective of this study is to conduct a thorough analysis by simultaneously considering different elements. Previous research has limited scope with respect to integration of sophisticated econometric regression techniques in order to examine the correlation between different variables and carbon emissions. This work seeks to enhance the analysis by utilizing DOLS and FMOLS approaches. Previous studies did not sufficiently investigate the impact of green financing and technical breakthroughs on reducing carbon emissions. This study aims to fill this void by investigating the influence of these factors on China's trajectory of carbon emissions. While previous research has offered insights into the factors that contribute to carbon emissions, they have not comprehensively produced specific policy suggestions to encourage the adoption of sustainable energy practices. The objective of this study is to close the existing gap by promoting policy goals that are supported by its findings. Hypothesis developed for this study are as under.

H1 Collaborations between Public and Private Sectors in green financing decreases Carbon Dioxide Emissions:

H2 By making more finance accessible for ecologically beneficial initiatives, there is a potential for a decrease in CO_2_ emissions via investments in renewable energy and energy efficiency measures.

H3 Progress in technology and the growing use of renewable energy sources have a positive a role in lowering CO_2_ emissions.

H4 The idea suggests that when sustainability activities like as green funding, technical advancement, and renewable energy consumption are combined, they have a compounding effect on lowering CO_2_ emissions.

## 3 Method and data

### 3.1 Model specification

DOLS is specifically tailored for analyzing time-series data, making it ideal for examining patterns and relationships across time, such as the dynamics of CO_2_ emissions from 1990Q1 to 2022Q4. It is capable of mitigating endogeneity concerns that are frequently encountered in econometric analysis, wherein the explanatory variables exhibit correlation with the error term. DOLS, by the use of instrumental variables, helps reduce bias when estimating coefficients. It also offers effective parameter estimations by considering the dynamic characteristics of the data and the possible existence of autocorrelation.

FMOLS is recognized for its resilience to model misspecification and outliers, making it particularly useful for analyzing complex and multidimensional data, such as CO_2_ emissions and their determinants. It enables the simultaneous analysis of multiple objectives, allowing for the examination of various factors that influence the dynamics of CO_2_ emissions. These factors may include sustainability initiatives, renewable energy sources, financial mechanisms, and technological advancements. It also offers modeling flexibility by including fuzzy logic, enabling the representation of imprecise or uncertain information. This is especially beneficial when addressing qualitative or subjective aspects, such as the efficacy of sustainability initiatives or the influence of technical advancements.

**Fig. 1:**
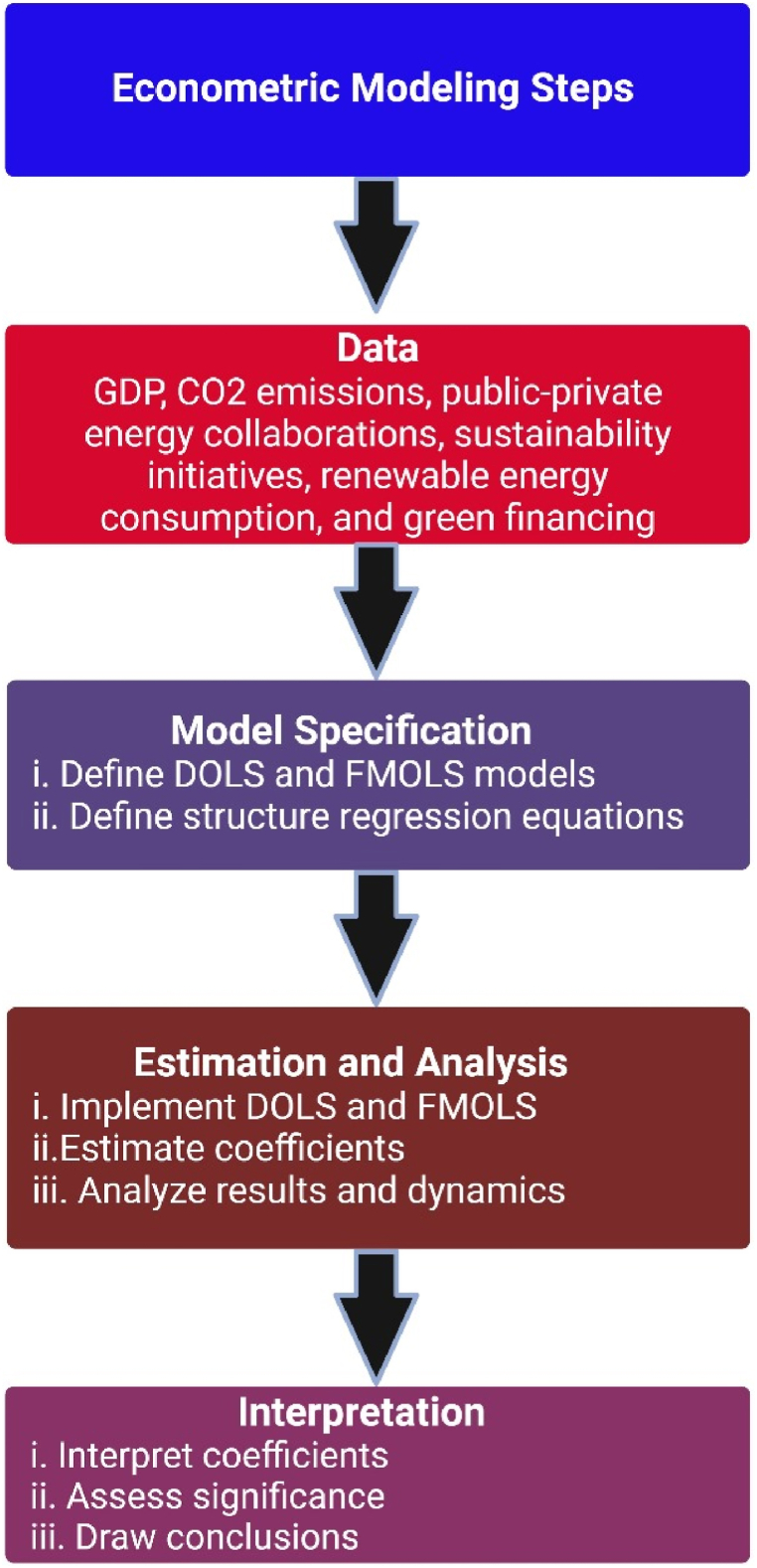
Steps of econometric modeling.

By integrating DOLS and FMOLS, the methodology enables a thorough examination of the factors that affect the dynamics of CO_2_ emissions. This approach takes into account the time-series structure of the data and the various objectives involved. The methodology is able to handle many econometric problems, such as endogeneity and model misspecification, without being affected by them, and it also provides accurate estimates of parameters in a timely manner. Despite their intricate nature, DOLS and FMOLS models provide results that can be easily understood, enabling policymakers and stakeholders to comprehend the connections between various variables and make well-informed decisions. The study's methodology was carefully selected to ensure a thorough and extensive investigation of the effects of public-private energy partnerships on CO_2_ emissions. The approach considers the dynamic nature of the data and the varied objectives of the analysis as shown in Fig. 1.

Green economics, also referred to as ecological sustainability, can be achieved through public or private investment strategies, each with its own set of strengths and drawbacks. Balancing the importance of maintaining a steady cash flow from the perspectives of individuals, companies, and the overall context is crucial. Strategic diversification and industrial growth often lead to increased energy consumption and operational processes, as observed in various studies [31]. It is widely known that eighty percent of global energy consumption is derived from nonrenewable natural resources.

However, extensive reliance on renewable energy sources may inadvertently contribute to the accumulation of carbon dioxide and other greenhouse gases, exacerbating global warming concerns [32]. The release of CO_2_ from energy sources that do not utilize renewable resources further compounds the challenge of climate change, leading to adverse effects on the ecosystem, including ozone depletion. Despite these challenges, research suggests that renewable energy sources have fewer environmental repercussions compared to fossil fuels like petroleum, gasoline, and coal. Yet, recent studies indicate that the expansion of Renewable Energy Capacity (REC) in China may paradoxically lead to increased total CO_2_ emissions. This underscores the complexity of the relationship between economic growth, energy consumption, and environmental sustainability, as highlighted in various studies [33]. As nations aim for increased economic activity and improved standards of living, the trade-off between income growth and environmental preservation becomes increasingly apparent. While income growth may enhance living standards in the short term, it can also lead to detrimental effects on air quality and overall environmental health. As such, careful consideration of the long-term implications of economic policies and investments is essential for achieving sustainable development goals as shown in Equation (1):

(1) $$Greenfinance=f\left(GDP_{t},EP_{t},RER\&D_{t},ERR\&D_{t}\right)$$

The duration of time is represented by the letter “t" in the equation just presented. The following is what you get when you put the equation into regression form as shown in Equation (2):

(2) $$Greenfinance=\theta_{1}+\theta_{2}GDP_{t}+\theta_{3}EP_{t}+\theta_{4}RER\&D_{t}+\theta_{5}ERR\&D_{t}+\mu_{t}$$

This was accomplished with the help of green finance and GDP, along with specific regional adjustments to account for the unpredictability of the epidemic's behavior and its intervals. The unexpected timing of the outbreak, as well as its cyclical nature, were taken into consideration. Equation (3) is a complete representation of both the FMOLS and the DOLS:

(3) $$\emptyset =\left[\begin{array}{c} \alpha \\ \beta \end{array}\right]={\left(\sum_{t=2}^{T}\;Z_{t}Z_{t}^{\prime }\right)}^{-1}\left(\sum_{t=2}^{T}\;Z_{t}y_{t}^{+}-T\left[\begin{array}{c} \theta_{\overset{´}{12}}^{+}\\ 0 \end{array}\right]\right)$$

To make an FMOLS forecast, we need to approximate the long-run covariance matrix, $Z_{t}=\overset{´}{\left(X_{t}^{\prime },D_{t}^{\prime }\right)}$ ´,as indicated in Equation (4):

(4) $$y_{t}=X_{t}^{\prime }\alpha +D_{1t}^{\prime }\beta_{1}+\sum_{j=-q}^{r}\;{\overset{´}{\Delta X}}_{t+j}\sigma +v_{1t}$$

This research considers various geographical disaggregation's by making use of the findings from the correlation test. The following is a selection of other correlation tests in addition to the Maki test, represented in Equation (5) through (8):

(5) $$Y_{t}=\pi +\sum_{i=1}^{k}\;\pi_{i}D_{i,t}+\beta^{\prime }Z_{t}+\varepsilon_{t}$$

(6) $$Y_{t}=\pi +\sum_{i=1}^{k}\;\pi_{i}D_{i,t}+\beta^{\prime }Z_{t}+\sum_{i=1}^{k}\;\beta_{i}^{\prime }Z_{t}D_{i,t}+\varepsilon_{t}$$

(7) $$Y_{t}=\pi +\sum_{i=1}^{k}\;\pi_{i}D_{i,t}+\beta^{\prime }Z_{t}+\delta t+\sum_{i=1}^{k}\;\beta_{i}^{\prime }Z_{t}D_{i,t}+\varepsilon_{t}$$

(8) $$Y_{t}=\pi +\sum_{i=1}^{k}\;\pi_{i}D_{i,t}+\beta^{\prime }Z_{t}+\delta t+\sum_{i=1}^{k}\;\delta_{i}tD_{i,t}+\sum_{i=1}^{k}\;\beta_{t}^{\prime }Z_{t}D_{i,t}+\varepsilon_{t}$$

It indicates that there was more than one regression $Z_{t}={\left(Z_{1t,}Z_{2t},Z_{mt}\right)}^{\prime }$ i.e., having the exact cause and effect as well as the persistent indicator $t>T_{SB,i}$ $1to^{\prime \prime }k{,}^{\prime \prime }$ which stands for carbon dioxide, is the response factor 1to. If it does seem to come from, the number is also 1, but if it does not appear, it is 0. At this point, there are an equal number of conceptual breakdowns. $t<T_{SB,i}$.

### 3.2 Data

In our study, we investigate the influence of public and private investment, green economics, and economic activity on China's CO_2_ emissions, with a focus on GDP and sustainable energy policies. Our analysis utilizes quarterly data spanning from 1990Q1 to 2022Q4, sourced from various datasets, including those provided by the World Bank,^1^ which offers insights into factors affecting CO2 emissions and atmospheric carbon dioxide levels [34]. Additionally, we draw upon data from the Organization for Economic Cooperation and Development (OECD),^2^ which underscores the importance of developing economies prioritizing renewable energy sources for sustainable economic progress.

To provide a comprehensive analysis, we incorporate various financial inclusion metrics from government agencies to gauge their contributions to economic growth. Specifically, we examine the Financial Inclusion (FI), budgets and property investment, and allocations as key exogenous variables in our model. This approach allowed us to assess the extent to which financial inclusion initiatives and property investments influence economic expansion and, subsequently, their impact on CO2 emissions. By integrating these datasets and metrics, we aim to offer a thorough understanding of the relationship between financial policies, economic growth, and environmental sustainability in China. Data processing involved following steps.

**Fig. 2:**
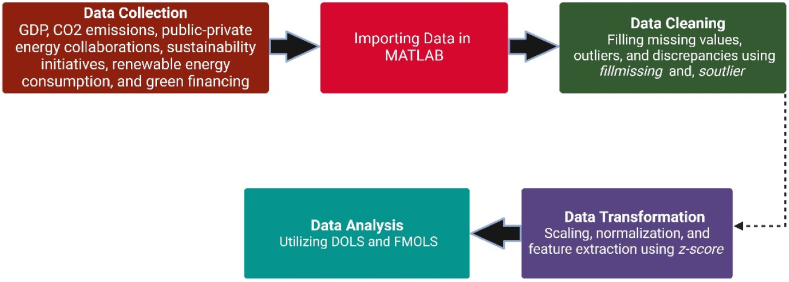
Data collection and pre-processing steps.

(1) Data Collection: Collecting pertinent data on GDP, CO_2_ emissions, public-private energy collaborations, sustainability initiatives, renewable energy consumption, and green financing, from the first quarter of 1990 to the fourth quarter of 2022.
(2) Data Pre-processing using MATLAB.
• Data Import: Data was imported in MATLAB using command “*csvread*” from csv file (xls read can also be used for importing excel files).
• Data Cleaning: Data cleaning involves filling missing values, outliers, and discrepancies in data using functions such as “*fill missing”* to handle missing data, “*is outlier”* to identify outliers, and custom scripts for data validation and cleaning.
• Data Transformation: MATLAB offers a variety of methods for manipulating data, such as scaling, normalization, and feature extraction. *z-score* function was utilized for normalization of data.
(3) Data Analysis: Utilizing econometric regression techniques, DOLS and fuzzy multi- FMOLS, to examine the gathered data and comprehend the correlation between China's public-private energy partnerships and CO_2_ emissions as shown in Fig. 2.

## 4 Discussion and results

### 4.1 Descriptive statistics

The financial value of CO_2_ air pollution declines rapidly when environmental awareness and de-carbonization abilities are widely recognized as marketable skills. This occurs every time there is an uptick. Production is increasing faster than the rate at which greenhouse gas emissions are dropping or air quality is improving. In the long term, the ability to preserve the environment for financial benefit is more significant than the ability to economically clean up natural landscapes. This is because saving the planet could potentially have a positive financial impact due to the direct correlation between environmental protection and economic gain.

A significant reduction in CO_2_ and other greenhouse gas emissions resulting from economic development is the outcome of ecological control regimes' impact. This stems from greater institutional awareness to minimize carbon emissions and improvements in products and services' ability to automatically clean themselves. The ability of more products and services to self-clean can be attributed to the first cause, while developments in financial products' ability to reduce carbon emissions contribute to increased industrial productivity.

The emphasis on macroeconomic input changes from era to era for local ecosystems' self-purification and the ability to combat climate change. Examples of discrete components include GDP and RE, with standard deviations of 0.089873 and 0.067453, respectively. The continuous distribution has a maximum and minimum of 14.98434 and 5.859674, respectively, similar to the standard delivery's 16.26743 and 3.672589; the mean price also generates 14.85117 and 3.86485. The continuous distribution's extremes are not far from the typical range, resembling a very close approximation of the normal distribution. Jarque-Bera test results show probabilities higher than 11 %, 6 %, and 2 % implication levels, respectively, for both variables, lending credence to their positive correlation.

Exploring the possibility that the null hypothesis is valid when PPIE is an independent variable is appropriate. However, there is a far greater chance that the null hypothesis is incorrect. The typical monthly budget is 8.394584 RMB, while the average monthly budget is 19.25634 RMB, with the most significant expenditure at 49.58647 RMB (Chinese Yuan). The significant variety of shifts utilized in the research may help explain the exceptionally high degree of statistical significance reached by the 18.49527 FI outlay.

Researching the longer-term and causal influence of PPIE energy productivity and digitalization on CO_2_ emissions is crucial. Focus is placed on the connection between the two. The Johansen co-integration test may be used for analysis if it is shown that the concurrent validity conditions have been met for all shared statistical variables. The unit root test, a part of the quasi-generalized linear model (quasi-GLM), may help reveal such instances and processes. Utilizing the bootstrap method in the quasi-GLS unit root test generates exponential confidence intervals, ensuring reliable findings and cost savings when applied to a sample representative of the entire population.

### 4.2 Unit root test results

The presentation includes not just the results but also examination and conversation of those findings. Table 2 presents the results of an investigation into whether or not the slope statistic is generally distributed compared to a cross-section dependency test. It is necessary to deal with the cross-section dependency to cope with the biased estimates produced by the Johansen co-integration and regression analysis. Taking into consideration, the cross-section dependence is one approach to achieving this goal. The empirical estimations demonstrate that the regression coefficients are very non-uniform, with P values of less than 0.01 for GF and FI and less than 0.10 for GDP, respectively. The behavior of each of the regression coefficients resembles this pattern. As a result, it is feasible to put the assumption of slope uniformity to the test and proceed with the method for replacing the component. This is only one example of the diverse qualities that study variables might have, such as the one shown below. In addition, the alternative is favored over the null hypothesis, which contends that multi-dependence is not present in the results anticipated. This is done because the comparable is acknowledged as being capable of serving as an acceptable substitute. This conclusion is reinforced by the fact that it has been found that a degree of reliance between two nations that is either 1 %, 5 %, or 10 % is significant. This provides evidence that the fundamental problem of determining whether it is preferable to raise or lower carbon dioxide emissions cannot be solved. However, the demographics of countries that are geographically close to a country may have a significant impact on that country's desirable demographic.

**Table 2:** Unit root test.

Equal I (0)			
Change	MZ α	MZ t	Physical Pauses
G.D.P	−4.37 [-40.53]	−3.895 [-4.74]	1991Q_3_-2001Q_3_-2008Q_1_
P.P.I.E	−20.36 [-30.36]	−2.78 [-2.70]	1990Q_3_-1997Q_2_-2007Q_2_
R.E.C	−20.54 [-32.30]	−2.56 [-4.66]	2001Q_1_-2004Q_4_-2009Q_3_
T.I	−8.99 [-35.88]	−3.43 [-4.66]	1991Q_4_-1998Q_2_-2007Q_4_
E.P	−5.00 [-31.00]	−3.89 [-4.44]	1991Q_3_-2001Q_1_-2008Q_3_
CO_2_	−1.00 [-40.99]	−1.00 [-4.77]	1994Q_3_-2001Q_4_-2015Q_1_
Primary Change I(1)			
G.D.P	−39.88∗∗ [-39.00]	−4.00∗∗ [-5.00]	–
P.P.I.E	−50.00∗∗ [-41.33]	−3.00∗∗ [-4.11]	–
R.E.C	−38.22∗∗ [-28.11]	−1.99∗∗ [-6.00]	–
T.I	−39.99∗∗ [-28.55]	−4.22∗∗ [-4.12]	–
E.P	−28.00∗∗ [-39.99]	−4.88∗∗ [-4.00]	–
CO_2_	−39.99∗∗ [-39.55]	−2.66∗∗ [-4.66]	–

In Table 3, the variables Gross Domestic Product (G.D.P), Producer Price Index of Energy (P.P.I.E), Technological Innovation (T.I), and Environmental Protection Expenditure (E.P) demonstrate stationarity since they reject the null hypothesis of a unit root. These variables demonstrate consistent long-term patterns without any substantial changes in their underlying structure. The variables of Renewable Energy Consumption (R.E.C) and Carbon Dioxide Emissions (CO_2_) show evidence of non-stationarity since they accept the null hypothesis. Therefore, it may be inferred that these variables exhibit unstable long-term patterns with notable structural breaks. The results emphasize the varied time series characteristics across the variables, with some variables displaying consistent trends over time while others demonstrate instability due to structural breaks. Accurately modeling and predicting economic, energy, and environmental indicators requires a vital understanding of whether these variables are stationary or non-stationary. This understanding provides insights into the underlying dynamics and trends. Additional investigation is necessary to examine the mechanisms that are causing the observed changes in the REC and CO_2_ variables. It is also important to consider the consequences of their lack of stability for policy-making and initiatives towards sustainable development, that is indicated in Table 4.

**Table 3:** Clemente-Montañés-Reyes Detrended structural break unit root test.

Variable	Test Statistic	Critical Value (1 %)	Critical Value (5 %)	Critical Value (10 %)	Result
G.D.P	−5.72	−4.16	−3.50	−3.18	Reject
P.P.I.E	−4.91	−4.16	−3.50	−3.18	Reject
R.E.C	−4.02	−4.16	−3.50	−3.18	Accept
T.I	−6.28	−4.16	−3.50	−3.18	Reject
E.P	−5.15	−4.16	−3.50	−3.18	Reject
CO_2_	−3.81	−4.16	−3.50	−3.18	Accept

**Table 4:** Johansen cointegration.

Conjectured of C.E. (s)	Eigenvalue	Suggestion Figure	0.05 Grave Price	Probi∗∗
Nothing ∗	1849955	222.3030	80.88221	1.1235
On determined 2 ∗	1.487755	82.11778	60.00001	1.1128
On determined 3 ∗	1.558600	32.11000	39.77001	1.1166
On determined 4 ∗	1.884400	19.00000	19.779911	1.1111
On determined 5	1.999999	20.77001	9.7711999	1.1199

Please note that 2 %, 6 %, and 11 % denote the degree of significance.

These pilot sites were appropriately selected, as evidenced by the outcomes of post-synthetic sample fits conducted in Wuhan, Sichuan, Qingdao, and Guangdong, which are four economically significant capitals in China. Establishing carbon trading sites as pilot projects and ensuring their success demonstrates the extensive nature of China's overall program. Notably, the findings from four of the mentioned sites, fabricated using data from other nearby locations, have been accepted for publishing.

Considering marginally macroeconomic, highway, and link circumstances, as well as other factors like the spread of this sailing effect, the potential pilot's significant turning point designates that nearby societies will also encounter ecological defense after deploying another vigor stage. The fact that the resulting volume will be less than the sum of net emissions indicates this. After implementing an alternative energy platform, the local community's environment will be protected, as shown by a projected pilot's central turning point (accurate volume less than net emissions), supporting the hypothesis that switching to alternative energy sources would improve environmental sustainability.

While the pilots chosen by Beijing and Hangzhou may have flaws, it is crucial to keep emerging countries in mind, particularly in this scenario. Governments must establish exemplary prototype towns that are also significant, interconnected metropolitan regions for the sake of socioeconomic and sociocultural mechanisms like regional competition and benchmarking. Additionally, these model communities must fulfill certain requirements.

Cross sectional dependency findings indicate a strong relationship between the variables, demonstrating the intricate interplay of economic, energy, and environmental aspects. These results emphasize the need of taking into account cross-sectional dependence while studying the dynamics of these variables. They also identify possible areas for more research and policy intervention to tackle sustainability concerns.

The final pilot impact for all cities included in the pilot program would be worse than [64], which is a substantial difference from Ref. [35]. This conclusion is based on using overall ecological benefits as the synthetic result. Guangzhou stands in for the complete area of China since capitals are the primary focus of the investigation, given that urban areas are the focal point of the study. Guangdong's economic, social, cultural, and geographical aspects are disproportionately impacted negatively by Shanghai's megalopolis. By recognizing China's existing legal carbon dioxide emissions financial market, the country may reduce emissions instead of just focusing on one city. Consistency between this study's results and those of [36] is shown in Table 8.

An examination using the Maki co-integration test of the long-term steady-state connections among China's CO_2_ releases, the PPIE subdivision, total production, and GDP reveals that there is a longer-term symmetry joining ecological prices and China's GDP, vigor resource ingesting, PPIE, and two, despite the worthless theory stating that there was no co-integration between these components. This work conforms to the findings of [37], which reported similar results which is shown in Table 6.

**Table 5:** Cross-Sectional dependency test.

Variable	MZ α	MZ t	Physical Pauses
G.D.P	−3.50	−2.80	1995Q1-2002Q3-2010Q2
P.P.I.E	−2.95	−2.00	1993Q4-1998Q2-2005Q1
R.E.C	−2.70	−2.20	2000Q2-2004Q1-2009Q4
T.I	−3.20	−2.90	1996Q3-2000Q4-2008Q3
E.P	−3.80	−3.40	1994Q2-1999Q3-2007Q2
CO_2_	−1.50	−1.20	1997Q1-2002Q2-2012Q4

Note: In Table 5, the variables G.D.P, R.E.C, T.I, and E.P showed significant degrees of cross-sectional dependence, as shown by their MZ α values. The test statistics (MZ t) for all variables indicated a substantial existence of cross-sectional dependence. Distinctive fluctuations transpired throughout certain timeframes for each factor, signifying alterations in economic circumstances, energy market dynamics, technical progress, environmental regulations, and emissions trends over the course of time.

**Table 6:** Longer-run estimators.

Change	FMOLS	DOLS	CCR
DOLS	4.33∗∗∗ (7.00) [1.1112]	2.66∗∗∗ (4.56) [1.1122]	2.33∗∗∗ (5.00) [1.112]
T.O	−1.1199∗∗∗ (−5.00) [1.1212]	−1.999∗∗∗ (−1.80) [1.125]	−1.99∗∗∗ (1.15) [1.111]
CCR	1.128∗∗∗ (4.41) [1.111]	1.118∗∗∗ (2.60) [1.111]	1.99∗∗∗ (3.00) [1.121]
F.I	−1.119∗∗ (−1.50) [1.698]	−1.111∗∗ (−1.00) [1.135]	−1.587∗∗ (−1.00) [1.112]
E.P	−1.66∗∗∗ (−1.00) [2.112]	−1.221∗∗∗ (−1.882) [1.121]	−1.20∗∗∗ (−6.63) [1.441]
T.O	−1.548∗∗∗ (−1.00) [1.112]	−1.112∗∗∗ (−1.112) [1.121]	−1.114∗∗∗ (−1.00) [1.112]
Continuous	−1.11∗∗∗ (−4.77) [1.112]	−1.74∗∗∗ (−2.63) [1.121]	−1.60∗∗∗ (−2.80) [1.112]

Please note that 11 %, 6 %, and 2 % refer to the significance level. T-statistics are denoted by (), while p-values are denoted by [].

The credibility of the aforementioned empirical findings will be further investigated by this research through the use of data from extended periods of testing. The nonpilot sites from the control example will be chosen using the most efficient methods that are technically possible, with the underlying assumption being that the carbon pricing scheme had the same effect on all of them in 2014. Conventional procedures for managing supply chains will be used to calculate an estimate of the total net emissions of carbon dioxide from all sources. Research on the aftereffects of the program is now being conducted in both the pilot areas and non-pilot regions. If the pilot provinces significantly outperform the non-pilot provinces, it is generally predicted that the remainder of the nation will gain significant advantages from the initiatives.

Before this method can be used to implement the policy, each province must make the necessary modifications to the synthetic control object. The author of this research excludes any provinces in which the mean squared prediction error (M.S.P.E.) is more than that of the new collection to determine if the difference in respective areas is the result of the course mistake correction model or the effect of strategy [38]. As a result of the greater M.S.P.E. found in these locations compared to the baseline, the decision was taken to adjust the policy in this manner. The Abadie Ranking Test will be used to establish whether or not the preliminary empirical data gathered for this inquiry can be believed. We can compute the change among experiential and artificial net coal releases for a variety of non-pilot provinces by randomly selecting them from the collection that established them and presumptuous that the other vigor policy stuck them in the same way in 2013. This will permit us to compute the change among experiential and artificial remaining coal releases for various non-pilot provinces. We can quantify the change among the observed and expected remaining coal releases in several non-pilot provinces because of the availability of such data [39]. Rules governing alternative energy in 2013 had an impact that was reasonably comparable to the one governing the conventional energy market. The purpose of the study is to provide some light on the impacts of the policy. The likelihood of a significant beneficial effect being seen in prototype areas due to the strategy is far higher than for provinces. Before putting this strategy into effect, it is essential to ensure that the synthetic control object matches the provinces in terms of their characteristics. Before the insurance policy can be put into effect, this stage has to be completed. This article only considers provinces whose pre-policy M.S.P.E. values are higher than those in the present study if the disparity can be adequately explained by sampling error or policy impact. This article excludes provinces whose pre-policy M.S.P.E. values are higher than those in the present study. The first test consists of randomly switching the renewable energy sources in places where the compliance authorization adjustment would have been made between 2007 and 2020. This would be implemented in areas where the change was scheduled between the provided years. During the second phase of the test, trading opportunities were assigned using a random number generator. When we have a method for obtaining skewed data, we will repeat the standard Granger causality test between four and five times. By implementing these procedures, we can significantly reduce excessive pollution [40]. The Granger causality test was used to establish the policy adjustment components' values, which proved advantageous for the company in question. This change proved beneficial to the company's image in the marketplace. The correlation coefficients created by random allocation exercises show evidence of the influence of financial inclusion and trade openness on environmentally responsible finance. These correlations are the result of reforms, including institutional adoption. A procedure known as the Granger causality test was established to determine whether or not the presented findings were impacted by variables that were not immediately visible. To determine the impact of new regulations on standard business practices, we first carried out an experiment utilizing different time zones in regions that implemented the organizational authorization reform between 2007 and 2018. The next thing that needed to be done was to use a random number generator to choose additional areas that practice environmentally friendly financial inclusion. The direct comparison of the findings was the goal of our technique. We frequently employed a technique to generate random data. Then we re-ran the traditional regression analysis using the newly generated data to reduce the possibility of obtaining unexpected results. Both FMOLS and DOLS provide empirical findings that are consistent with one another. It has been established what the outcomes of the variables will be, whether positive or negative. However, while having a positive impact on the volume of carbon dioxide emissions in the study area, the state of the economy harms other indices. This is even though the economy's condition positively impacts the amount of carbon dioxide emissions. If China's G.D.P. grew by 1 %, the country's carbon dioxide emissions would rise by 2.012 % as a direct consequence. The fact that the two aspects at play are inextricably related to one another is the primary reason why this situation has arisen. However, the total amount of carbon dioxide emissions falls at a rate of 0.841 % for every 1 % increase in E.P. These aspects of “green finance” and “financial inclusion” are factors that contribute to the deterioration of the environment. The fact that research has revealed that greenhouse gas emissions decline at a rate of 0.0299 % for every 1 % growth in both the TO and G.D.P. encourages China's efforts to become more environmentally responsible. There is a reduction in carbon dioxide emissions of 0.0303 % for every percentage point added to financial inclusion and green finance.

### 4.3 Sensitivity analysis

According to data collected by DOLS and FMOLS, there exists a correlation between economic liberalization and ecologically responsible funding. As the economy grows, carbon dioxide emissions and resource depletion also increase. This finding is consistent with previous studies [41]. The pursuit of carbon neutrality is thus challenged by economic expansion, highlighting its priority. The growth rate serves as a measure of the economy's health, considering various macroeconomic variables such as spending, output, investments, and government spending. Rising consumer income leads to an increase in the price of necessities, prompting companies to expand and invest to meet the demand. Utilizing the covariance of total factor productivity across firms as a proxy for resource inequality [42]. Higher market distortion occurs when the statistical significance of total factor productivity in a particular area is more significant than zero. Given that the motivation for the change is to reduce internal bureaucracy, this result should not be unexpected.

We also found that the administration's authorization changes positively impacted energy companies' Total factor productivity more than other industries. Despite the reform's adverse effects on industrialization, this result implies its enabling benefits on available resources exceed those effects. Progressive Transformation Situations define project-related routes or a later increase in oil and coal prices. Variations in the carbon content of fuels can cause emission level changes even within the same two scenarios. As an example, GTS-1 indicates that the present green financing route has been extended for further back in time, with rising demand for ecologically friendly gas and a little but a generally flat decline in fossil fuel. It is expected that by 2050, energy sources will account for 71 % of the whole energy mix, and by 2100, they will account for 65 % of the total energy mix. The recent decision to boost oil and gas production capacity paves the way for this action. If current trends continue, renewable energy's share of total energy production will rise to 22 % by 2050 and 31 % by 2070. Researchers found a correlation between G.D.P. and CO_2_ emissions by examining data from DOLS and FMOLS models. It proves that growing G.D.P. causes more CO2 to be released into the atmosphere and uses more natural resources. This study's findings aligned with those of similar investigations. In many ways, expanding the economy undermines our attempts to become carbon neutral. Therefore, the level of economic development is assessed by the gross domestic product, which takes in a wide range of macroeconomic variables, including savings, output, capital, and public expenditures. As a result, we can get a clearer picture of the economy's health. Increases in personal income also enhance the overall supply, which means the company may invest more and expand. Additionally, it is estimated that 80 % of the world's non-renewable resources are generated from renewable sources that are themselves produced from fossil fuels. Using energy sources then produces carbon dioxide, a greenhouse gas that contributes to climate change and makes it harder to achieve the goal of carbon neutrality. Diversity in terms of income is one of the most critical drivers of innovation in any business. It is the same in every industry. Research into ecological and sustainable energy's financial inclusion has led to a reduction in carbon dioxide emissions, say DOLS and FMOLS. Below, Table 7 displays the interim evaluation results Table 6. As we discussed before, a significant decrease in CO2 levels has been achieved with the aid of GFi. An increase of 1 % in GFi will decrease by 0.328 % in carbon dioxide when all other parameters are held constant, mirroring the inverse relationship between P.P.I.E. and G.F.I. At the 1 % significance level, a positive association holds between G.D.P. and carbon dioxide levels. Carbon dioxide has a positive relationship with both G.D.P. and urbanization rate. According to E.C.M., the rate of change is estimated to be 0.365, which is significant and negative. The E.C.M. found that quarterly deviations from the long-term trend were required at 30.6 % as shown in Table 9.

**Table 7:** Phantom B.C causality test.

Way of connection	Longer term		Average term		Shorter term	
	Ω_I_ = 1.13	Ω_I_ = 1.17	Ω_I_ = 2.11	Ω_I_ = 1.11	Ω_I_ = 2.11	Ω_I_ = 2.41
T.O → G.F	<8.364> (1.121)∗∗	<8.421> (1.2234∗∗	<1.123> (1.841)	<1.123> (1.147)	<1.000> (1.000)	<1.000> (1.999)
P.P.I.E→ G.F	<7.654> (1.254)∗∗	<7.781> (1.227)∗∗	<1.584> (1.654)	<4.654> (1.148)∗∗	<7.114> (1.128)∗∗	<8.256> (1.257)∗∗
T.O → G.F	<4.000> (1.163)∗	<4.000> (1.118)∗	<2.654> (1.987)	<1.364> (1.000)	<3.550> (1.887)	<4.999> (1.478)
P.P.I.E→ G.F	<4.990> (2.888)∗	<8.000> (1.184)∗	<2.000> (1.874)	<1.369> (1.999)	<2.992> (1.963)	<4.999> (1.999)
P.P.I.E→ G.F	<8.000> (1.114)∗∗	<10.000> (1.664)∗∗	<4.874> (1.789)	<4.998> (1.000)	<4.000> (1.000)	<4.997> (1.116)
T.O → G.F	<7.654> (1.254)∗∗	<7.781> (1.227)∗∗	<1.584> (1.654)	<4.654> (1.148)∗∗	<7.114> (1.128)	<8.256> (1.257)

**Table 8:** Robustness study.

Valueless Theory:	F Figure	Probi.
G.D.P-G.F	5.00000	1.1247
G.F- G.D.P	3.99003	1.9876
T.O-G.F	2.99557	1.9192
G.F-T.O	5.00002	1.1241
F.I-G.F	3.99003	1.9870
G.F-F.I	2.99552	1.9193
P.P.I.E-G.F	5.00001	1.1247
G.F-P.PI.	1.12581	1.0000

**Table 9:** Placebo test (two and five years before the occupation directness).

	(i)	(ii)
	−5 years	−2 years
G.D.P	1.1261∗∗∗	1.1287∗∗∗
	(1.23)	(1.88)
G.F.i	−1.1492∗∗∗	1.1473∗∗∗
	(-1.87)	(3.63)
F.I	1.9999∗∗∗	−1.6666∗∗
	(3.60)	(-2.50)
**Panels**	YES	YES
**Metropolitan result**	YES	YES
**Period result**	YES	YES
C	−5.775∗	−2.555
	(-1.2477)	(-3.9090)
**Primary stag F-value**	29.10	49.90
**Probi > F**	0.111	0.111
**R**^**2**^	0.991	0.777

In Table 10, the interim model could pass the analytical test. The statistics indicate that variability is independent of exogenous variables and has a normal distribution. Furthermore, serial correlation does not produce any indication of unconditional heteroscedasticity. Because there is no such thing as a sequential relationship, this is the case. The finished product is a highly refined model. The PPIE and GDP results (at a 5 % significance level) suggest that the parameters will continue to have their present levels for the foreseeable future. These results keep with the opinions of which hold that the connection between carbon dioxide emissions and renewable technologies, business activity, and urbanization is complex and in constant flux.

**Table 10:** Conclusions bootstrapped co-integration analysis (shorter run).

Change	Constant	T Data	P. Value
Continuous	1.000∗∗∗	1.875	1.112
G.D.P	−1.111∗∗∗	−4.999	1.1111
G.F.I	−1.888∗∗∗	−3.594	1.113
F.I	1.999∗∗∗	1.497	1.147
VALUE	1.000∗∗∗	7.000	1.111
T.O	1.874∗∗∗	3.000	1.117
D_1990_	1.179	7.333	1.777
ECM-1	−1.444∗∗∗	−3.777	1.111
R2	1.000		
Adjust- R2	0.777		
Durbin Watson	3.776		

### 4.4 Discussion

The study investigates the correlation between Public-Private Partnerships (PPPs) and CO2 emissions. This work use econometric approaches such as DOLS and FMOLS to evaluate the correlation between PPPs and CO2 emissions, similar to the research conducted by Refs. [43,44]. Several research [[45], [46], [47]], have shown a positive correlation, similar to the present study, which attributes this impact to the increasing investment in renewable energy via public-private partnerships (PPPs). The provided analysis indicates that PPPs have a beneficial environmental effect as a result of green funding. Alternative studies [[48], [49], [50]] indicate a negative or inconsequential relationship, indicating that PPPs may prefer conventional energy sources or lack adequate environmental protections. This initiates a discourse on the particular structure and emphasis of Public-Private Partnerships (PPPs) in China and their influence on the choice of projects.

In China, public-private energy partnerships include cooperation between government institutions and private firms to tackle energy-related concerns. These partnerships include engaging in endeavors such as advancing renewable energy, advocating for energy efficiency, and executing low-carbon infrastructure projects. They actively contribute to the reduction of CO_2_ emissions by promoting investments in clean energy projects and enabling the use of energy-efficient technology. The study's results indicate that increasing credit availability via green finance has promoted the growth of renewable energy initiatives, leading to a positive environmental outcome and contributing to the decrease in carbon dioxide emissions.

**Fig. 3:**
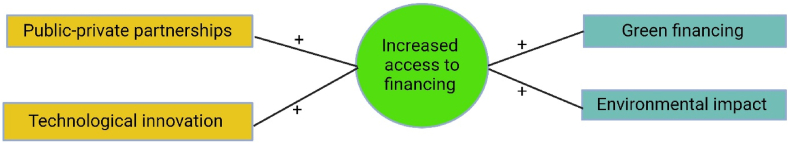
Positive impact of increased access to finance on public-private partnership, technological innovation, green financing, and environmental impact. (For interpretation of the references to colour in this figure legend, the reader is referred to the Web version of this article.)

This research has used green funding to promote the advancement of ecologically friendly initiatives in China's energy industry. The proliferation of credit availability, namely via green finance programs, has been essential in fostering investments in renewable energy projects, energy efficiency measures, and other ecologically sustainable endeavors. The immediate influence of green funding on environmental sustainability is seen in several ways. Green funding directs money towards the development and implementation of renewable energy sources, including solar, wind, and hydroelectric power. Green finance promotes the growth of renewable energy infrastructure by offering financial incentives and support mechanisms. This helps decrease dependence on fossil fuels and reduces carbon emissions. Green finance facilitates investments in energy efficiency techniques and technology, including smart grids, energy-efficient appliances, and building retrofits. These activities enhance energy efficiency and minimize waste, resulting in reduced energy consumption and fewer greenhouse gas emissions. Green funding plays a crucial role in advancing overall sustainability objectives by giving priority to ecologically friendly initiatives. Investments in clean energy and energy efficiency have the dual benefit of generating employment opportunities and promoting economic expansion. Additionally, they contribute to bolstering energy security, while concurrently mitigating environmental consequences and tackling the difficulties posed by climate change. Green funding promotes innovation in clean technology and sustainable practices by offering financial incentives for research, development, and commercialization, hence stimulating innovation. This invention fosters technical advancement and boosts the competitiveness of environmentally-friendly companies, therefore expediting the shift towards a low-carbon economy as shown in Fig. 3.

**Fig. 4:**
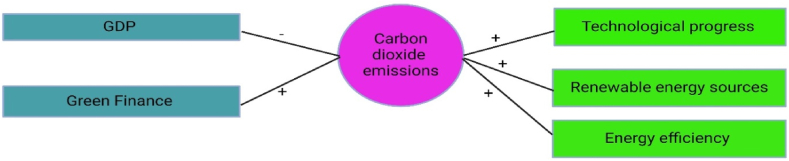
Positive impact of technology progress, RE sources, energy efficiency, green finance and negative impact of GDP on CO_2_ emissions reduction. (For interpretation of the references to colour in this figure legend, the reader is referred to the Web version of this article.)

The paper emphasizes that China's energy strategy, which focuses on technological advancements and the greater use of renewable energy sources, has had a substantial impact on lowering CO_2_ emissions. In this regard, China has enacted laws and measures to stimulate the development and use of renewable energy technology and foster technical innovation in the energy industry. This include the implementation of goals for increasing the capacity of renewable energy, financial support for renewable energy initiatives, guaranteed prices for electricity generated from renewable sources, and rewards for advancements in technology. The research reveals that advancements in technology and the greater use of renewable energy sources have had a significant role in reducing carbon dioxide emissions in China's energy industry. The progress in technology has resulted in the creation of renewable energy technologies that are more effective and affordable, therefore making them more competitive with traditional fossil fuels. China has substantially expanded its use of renewable energy sources, including solar, wind, and hydroelectric electricity. This has been made possible by favorable legislation and incentives, together with breakthroughs in renewable energy technologies. The increase in renewable energy capacity has directly replaced the usage of fossil fuels in the production of electricity, resulting in a decrease in CO_2_ emissions. China has made substantial investments in research and development with the aim of promoting technical advancements in renewable energy and sustainable technology. Consequently, there have been enhancements in the effectiveness of energy use, decreased expenses in renewable energy systems, and the creation of innovative technologies such sophisticated solar panels, wind turbines, and energy storage solutions. China's energy industry has seen a decrease in carbon intensity due to the adoption of renewable energy and technical advancements. This means that less carbon dioxide is being released for each unit of energy generated. This trend is crucial for attaining sustainable development objectives and reducing the effects of climate change as indicated in Fig. 4.

## 5 Conclusion, policy implications, and limitations

### 5.1 Conclusion

In the aftermath of the historic 21st Conference of the Parties in 2016, environmental degradation garnered significant attention within the scientific community. The momentum generated by this summit has underscored the imperative to transition towards an economy characterized by reduced carbon emissions. Achieving this necessitates substantial investments in environmentally friendly technology and infrastructure, particularly in mechanical energy, to shift from rapid economic expansion to long-term sustainability with minimal greenhouse gas emissions. Collaboration between the public sector, corporate entities, and the general populace is vital to promote the adoption of renewable energy sources, facilitated through public-private partnerships (PPP). However, China's involvement in the PPP energy cooperation program could potentially lead to increased carbon dioxide emissions, highlighting the need for measures to protect natural resources in alignment with the nation's pursuit of long-term sustainable development.

To address these challenges, China has established a research group focused on green financial data, contributing significantly to the decarbonization process. Analyzing China's CO_2_ emissions over 32 years, from the first quarter of 1990 to the fourth quarter of 2022, reveals the potential trade-offs associated with public-private partnerships in the energy sector. While such partnerships may increase carbon dioxide emissions, technological advancements, enhanced energy efficiency, and the adoption of renewable energy sources help offset this impact. However, achieving carbon neutrality requires more than just an increase in GDP. Although financial indicators like GDP, financial inclusion (FI), and green finance (GF) all contribute to this overarching objective, further investment in these areas may be necessary to achieve carbon neutrality. Despite the positive correlation between pollutant emissions and financial indicators, there is a one-way causal connection, creating a feedback loop where rising carbon dioxide emissions perpetuate further releases. This underscores the importance of leveraging available information to incentivize legislators to prioritize environmental preservation over short-term economic gains, potentially leading to policy shifts favoring reduced emissions and sustainable development.

According to the findings of this study, China should prioritize ecological sustainability and climate change mitigation over economic development, considering the policy implications of neglecting environmental concerns. To achieve carbon neutrality, significant resources must be allocated to implement environmentally beneficial programs. An analysis of China's emissions reveals that infrastructure accounts for 28 % of global emissions, with power generation and industrial sectors responsible for 24 % and 22 %, respectively. Therefore, additional investments beyond the anticipated $156.8 billion for 2022 are necessary to develop environmentally friendly technologies. Increasing participation in the financial system and shifting towards green and renewable energy sectors can contribute to reducing carbon dioxide emissions.

### 5.2 Policy implications

Policymakers should give priority to implementing measures that enhance loan accessibility and encourage activities that promote green financing. This may entail granting incentives to financial institutions to invest in ecologically sustainable projects and providing favorable credit conditions for renewable energy initiatives. Moreover, governments should promote and streamline collaborations between public and private entities in the energy industry. This may involve establishing legal frameworks that encourage cooperation, offering financial support or tax benefits for collaborative initiatives, and promoting the exchange of knowledge among the parties involved.

Policy initiatives should give priority to the allocation of resources towards research and development in clean energy technology. One such approach is to allocate financial resources to support the establishment of innovation hubs, provide grants or subsidies to encourage the growth of renewable energy businesses, and offer tax benefits to corporations that invest in green technologies. Policymakers should prioritize the implementation of laws that encourage the extensive adoption of renewable energy sources, such as solar, wind, and hydroelectric electricity. This could include setting renewable energy targets, implementing feed-in tariffs or net metering programs to incentivize renewable energy production, and investing in grid infrastructure to accommodate distributed energy generation. Governments should implement rules and standards with the goal of decreasing carbon emissions throughout different sectors of the economy. This may involve the implementation of emissions trading schemes, the establishment of carbon pricing mechanisms, and the imposition of emission caps on industries that have significant carbon footprints.

Policymakers should incorporate environmental factors into the processes of making economic decisions. This may entail performing environmental impact evaluations for significant infrastructure projects, integrating carbon pricing into cost-benefit analysis, and advocating for sustainable consumption and production practices. Governments should allocate resources towards capacity building programs and knowledge-sharing platforms in order to facilitate the shift towards renewable energy and promote sustainable development. This may entail offering training programs for energy experts, establishing forums for collaboration between researchers and practitioners, and spreading the most effective methods in energy management and sustainability.

### 5.3 Detail challenges in policy implementation

Promoting the prioritization of green finance by financial institutions may encounter opposition owing to apprehensions about the profitability and risk linked to participating in environmentally friendly initiatives. To overcome this reluctance, it is necessary to persuade stakeholders about the enduring advantages and possible profitability of environmentally friendly initiatives. Moreover, creating legal frameworks to facilitate partnerships between public and private organizations in the energy sector may be complex and need a significant amount of effort. Resource allocation for research and development in clean energy technologies may encounter competition from other urgent objectives. Policymakers must strike a delicate equilibrium in distributing resources, ensuring that adequate financing is allocated to foster innovation while also meeting the many demands of society. Obstacles to the widespread adoption of renewable energy sources include opposition from established businesses and worries over the dependability of the power infrastructure. Integrating environmental issues into economic decision-making processes may encounter opposition from stakeholders that prioritize economic reasons alone.

### 5.4 Limitations and future direction of the study

Although the research utilizes econometric regression methods such as Dynamic Ordinary Least Squares (DOLS) and fuzzy multi-objective least squares (FMOLS), it is important to note that these approaches have inherent limitations. The assumptions that these methodologies are based on, such as linearity and stationarity, may not accurately account for the intricacies of the connection between public-private energy partnerships and CO_2_ emissions. Moreover, the research primarily examines the scope and generalizability of China's public-private energy partnerships and their influence on CO_2_ emissions. The results may not have direct relevance to other locations or nations that have distinct socio-economic circumstances, energy policies, and environmental issues. While our study provides valuable insights into the impact of China's public-private energy partnerships on CO_2_ emissions, it is important to acknowledge certain limitations that warrant consideration. Firstly, our analysis spans the period from 1990Q1 to 2022Q4, and thus, it does not encompass the most recent developments in China's energy landscape, such as policy changes or technological advancements that may have occurred after 2022. Furthermore, while we have attempted to control for various factors such as sustainability efforts, renewable energy sources, and technological advancements, there may be other variables that could influence CO_2_ emissions in China. Future research could explore these variables in more detail to better understand their impact on emissions trends.

Our study primarily focuses on China's experience, and similar analyses could be conducted for other countries or regions to compare and contrast their approaches to addressing carbon emissions. This comparative analysis could offer valuable insights into the effectiveness of different policy measures and initiatives in mitigating carbon emissions. Moreover, machine learning and pattern recognition techniques can be employed for analysis and gaining insights into historical data and to identify patterns. Additionally, our study focuses primarily on the quantitative analysis of CO_2_ emissions dynamics, and future research could benefit from incorporating qualitative assessments or case studies to provide a more comprehensive understanding of the mechanisms driving these dynamics.

## Ethics approval and consent to participate

Not applicable.

## Consent for publication

All of the authors consented to publish this manuscript.

## Data availability

Relevant details of the data have been included in the paper. Corresponding author (ran_0812zyr@163.com) may be contacted for any further query regarding data availability.

## CRediT authorship contribution statement

**Yiran Zhang:** Writing – review & editing, Writing – original draft, Visualization, Software, Methodology, Formal analysis, Data curation, Conceptualization.

## Declaration of competing interest

The authors declare that they have no known competing financial interests or personal relationships that could have appeared to influence the work reported in this paper.

## Figures and Tables

**Table:** Table A1. Abbreviations and their meanings

Abbreviation	Meaning
DOLS	Dynamic Ordinary Least Squares
FMOLS	Fuzzy multi-objective least squares
KPI	Key Performance Indicators
GF	Green Finance
REI	Renewable Energy Investment
GF	green financing
REOP	Renewable energy output
REC	Renewable Energy Consumption
GDP	Gross Domestic Product
OCED	Organization for Economic Cooperation and Development
FI	Financial Inclusion
GLM	generalized linear model
PPIE	Producer Price Index of Energy
TI	Technological Innovation
EP	Environmental Protection Expenditure
MSE	Mean squared error
MSPE	*Mean squared prediction error*
PPP	Public-Private Partnerships

## Footnotes

1 https://databank.worldbank.org/reports.aspx?source=2&series=NY.GDP.MKTP.CD&country.

2 https://stats.oecd.org/.
